# Atrial natriuretic peptide stimulates autophagy/mitophagy and improves mitochondrial function in chronic heart failure

**DOI:** 10.1007/s00018-023-04777-w

**Published:** 2023-04-26

**Authors:** Salvatore Raffa, Maurizio Forte, Giovanna Gallo, Danilo Ranieri, Simona Marchitti, Damiano Magrì, Marco Testa, Rosita Stanzione, Franca Bianchi, Maria Cotugno, Emiliano Fiori, Vincenzo Visco, Sebastiano Sciarretta, Massimo Volpe, Speranza Rubattu

**Affiliations:** 1 Department of Clinical and Molecular Medicine, School of Medicine and Psychology, Sapienza University, Rome, Italy; 2 IRCCS Neuromed, Pozzilli, Isernia Italy; 3 Cardiology Unit, Azienda Ospedaliero-Universitaria Sant’Andrea, Rome, Italy; 4 Department of Medical-Surgical Sciences and Biotechnologies, Sapienza University of Rome, Latina, Italy; 5 IRCCS S. Raffaele, Rome, Italy

**Keywords:** Cardiac natriuretic peptide, Left ventricular systolic function, ARNi, Mitochondrial membrane potential, Mitochondrial oxidative stress, Autophagosome, Circulating mononuclear cells

## Abstract

Mitochondrial dysfunction, causing increased reactive oxygen species (ROS) production, is a molecular feature of heart failure (HF). A defective antioxidant response and mitophagic flux were reported in circulating leucocytes of patients with chronic HF and reduced ejection fraction (HFrEF). Atrial natriuretic peptide (ANP) exerts many cardiac beneficial effects, including the ability to protect cardiomyocytes by promoting autophagy. We tested the impact of ANP on autophagy/mitophagy, altered mitochondrial structure and function and increased oxidative stress in HFrEF patients by both ex vivo and in vivo approaches. The ex vivo study included thirteen HFrEF patients whose peripheral blood mononuclear cells (PBMCs) were isolated and treated with αANP (10^–11^ M) for 4 h. The in vivo study included six HFrEF patients who received sacubitril/valsartan for two months. PBMCs were characterized before and after treatment. Both approaches analyzed mitochondrial structure and functionality. We found that levels of αANP increased upon sacubitril/valsartan, whereas levels of NT-proBNP decreased. Both the ex vivo direct exposure to αANP and the higher αANP level upon in vivo treatment with sacubitril/valsartan caused: (i) improvement of mitochondrial membrane potential; (ii) stimulation of the autophagic process; (iii) significant reduction of mitochondrial mass—index of mitophagy stimulation—and upregulation of mitophagy-related genes; (iv) reduction of mitochondrial damage with increased inner mitochondrial membrane (IMM)/outer mitochondrial membrane (OMM) index and reduced ROS generation. Herein we demonstrate that αANP stimulates both autophagy and mitophagy responses, counteracts mitochondrial dysfunction, and damages ultimately reducing mitochondrial oxidative stress generation in PBMCs from chronic HF patients. These properties were confirmed upon sacubitril/valsartan administration, a pivotal drug in HFrEF treatment.

## Introduction

HF is a clinical syndrome affecting more than 60 million patients worldwide with a high associated morbidity and mortality [1].

Oxidative stress produced by both mitochondrial and cytosolic sources plays a pivotal role in the onset and progression of HF [2] alongside with chronic inflammation and energy deficit. In this context, the role of mitochondrial dysfunction appears of increasing importance [3]. An overproduction of mitochondrial ROS was documented in animal models with left and right ventricular HF induced by pressure overload [4, 5]. A mitochondrial dysfunction was reported in dilated cardiomyopathy and in failing hearts [6, 7]*.*

ANP, secreted by cardiomyocytes in response to stressors such as pressure or volume overload, is a critical regulator of cardiovascular homeostasis by promoting diuresis, natriuresis, and vasodilation [8]. It also plays important autocrine and paracrine functions within the heart and the vascular system [9, 10]. In the specific, ANP counteracts cardiomyocyte hypertrophy, reduces cardiac fibrosis, and promotes vascular integrity. We recently demonstrated for the first time that ANP exerts a cardioprotective effect through the NPR-A/PKG/TFEB signaling pathway regulating autophagy both in vitro and in vivo approaches [11].

Autophagy is a mechanism of self-defense in response to stress conditions devoted to the removal of dysfunctional intracellular elements, including whole organelles [12, 13]. Mitophagy is the selective form of autophagy devoted to the digestion of dysfunctional or irreversibly damaged mitochondria [14]*.* Compelling evidence showed a mechanistic link between reduced autophagy and increased cardio- and cerebrovascular disorders [15]. Interestingly, we recently showed that PBMCs from HFrEF patients have functionally and structurally altered mitochondria due to the inability to activate an effective antioxidant response and an adequate mitophagic flux [16].

Several strategies aimed to reactivate autophagy and to remove damaged mitochondria successfully reduced disease progression in preclinical models [17, 18]. The potential beneficial effects of ANP on autophagy activation in human diseases are currently unclear [19]. Based on our recent evidence, we hypothesized that an increase of ANP may represent a valuable strategy to activate autophagy and promote protection in conditions of autophagy dysregulation such as that observed in HF patients.

To this aim, we evaluated the ability of αANP to counteract the mitochondrial dysfunction through a potential strengthening of the autophagic response in PBMCs isolated from HFrEF. We applied both an ex vivo approach through the exposure to αANP of PBMCs from HF patients and an in vivo approach by isolating PBMCs of HF patients upon treatment with ARNi, the latter being known to increase circulating αANP level in HF patients [20, 21].

## Materials and methods

### Study subjects

We examined a total of nineteen consecutive HFrEF patients referring to the outpatient clinic of the Cardiology Unit of St. Andrea Hospital in Rome from April 2019 to March 2023. Out of them, for the ex vivo study, we enrolled thirteen patients matching the following inclusion criteria: age under 75 years and left ventricle ejection fraction (LVEF) below 40%. For the in vivo study, we recruited six HFrEF patients with similar characteristics and evaluated them before and after 2 month treatment with ARNi (sacubitril/valsartan) started at the dosage of 49/51 mg twice daily, rapidly uptitrated at the dosage of 97/103 mg twice daily, while maintaining unchanged the remaining therapy. LVEF and all other echocardiographic parameters were measured with 2D echo Simpson’s biplane during mono-dimensional and bi-dimensional echocardiography (Siemens Acuson C200, Erlangen, Germany). All patients included had HF signs and/or symptoms (New York Association functional class II–III, stage C of the American College of Cardiology/American Heart Association classification). Patients with recent hospitalizations for acute HF or other acute conditions within the last 3 months before the enrollment, with malignancy, inflammatory or infectious diseases, diabetes mellitus, history of cigarette smoking, and alcohol abuse were excluded. Three blood donors referring to the transfusion center of the same hospital were recruited as CTR.

### PBMCs preparation and cultures

Venous blood specimens were obtained from all HF patients and from healthy subjects (CTR). Samples were drawn into collection tubes containing EDTA, delivered directly to the Ultrastructural Pathology Lab of St. Andrea Hospital and immediately processed to isolate PBMCs using density gradient centrifugation over Ficoll-Paque^™^ PLUS (Amersham Biosciences/GE Healthcare).

For the ex vivo study, PBMCs were cultured in duplicate at the density of 1 × 10^6^ cells/ml on T25 flasks (Becton Dickinson, Oxnard, CA, USA) in RPMI-1640 supplemented with 10% fetal bovine serum and antibiotics (LPS; Sigma Chemicals Co.; St. Louis, MD, USA), treated from 4 h or not with αANP synthetic peptide (Primm, Naples, Italy) dissolved in PBS and diluted in complete medium at the final physiological concentration of 10^–11^ [11]. PBMCs were finally split into several aliquots for flow cytometry, RT-PCR assay, and ultrastructural evaluation.

For the in vivo study, the isolated PBMCs were directly analyzed at both pre- and post-treatment with sacubitril/valsartan.

### Evaluation of oxidative stress and mitochondrial membrane potential (Δψm)

For mitochondrial membrane potential (Δψm) assessment, the cells were incubated with 5, 5′, 6, 6′-tetrachloro-1, 1′, 3, 3′-tetraethylbenzimidazolylcarbocyanine iodide 1.5 μM (JC-1, Molecular Probes, Invitrogen, Eugene, OR, USA) for 30 min at 37 °C. The samples were protected from light, washed with warmed PBS, re-suspended in pre-warmed medium, and collected with MACSQuantH Analyzer flow cytometer (Miltenyi Biotec GmbH). The excitation and the emission wavelengths were 488/525 to 585 nm (B1 + B2 channels) for JC-1 polychromatic fluorescence emission evaluation.

For intracellular ROS detection, the cells were incubated with 5 μM 2′,7′-dichlorofluorescein diacetate (DCFH-DA, Sigma-Aldrich, St. Louis, MO, USA) for 10 min at 37 °C for cytoplasmic oxidative stress assessment, or with 1 μM MitoSOX^™^ Red mitochondrial superoxide indicator (Molecular Probes, Invitrogen) for 15 min at 37 °C for mitochondrial oxidative stress assessment, protected from light, extensively washed with PBS, re-suspended in pre-warmed medium, and collected with MACSQuantH Analyzer flow cytometer. The excitation and the emission wavelengths were 488/525 nm (B1 channel) for DCFH-DA and 488/585 nm (B2 channel) for MitoSOX^™^ Red detection. The cellular response to oxidative stress was assessed by the analysis of the expression of p66shc mRNA, a marker of mitochondrial stress and of ROS-induced ROS production.

### Autophagy and mitophagy assessment

Autophagy was assessed by the analysis of the expression of LC3 and Beclin mRNA, two established markers of autophagic pathway activation [22, 23] and by cytofluorimetric assay with LysoTracker dye (LysoTracker Red, Molecular Probes, Eugene, OR; 50 nM for 30 min at 37 °C), that allows the simultaneous evaluation of an autophagy-related process and of other functional assessment in the living cells [24, 25]. The presence and the characterization of autophagic structures within the cells were obtained by TEM [26].

Mitophagy was evaluated by cytofluorimetric assessment of mitochondrial mass upon staining with MitoTracker Green (MTG-FM; Molecular Probes, Invitrogen; 50 nM for 30 min at 37 °C) [27, 28] and by colocalization analysis of fluorescent probes MitoTracker Green and LysoTracker Red (see above for staining details). Fluorescence signals were acquired by ApoTome System connected with an Axiovert 200 inverted microscope (Zeiss, Oberkochen, Germany). The quantitative analysis of the extent of colocalization was performed by Axiovision software (Zeiss) equipped with colocalization module.

To examine the pathways of mitophagy, the gene expression of *Parkin* (for the ubiquitin-related mitophagy), *BNIP3*, and *FUNDC1* (for the receptor-mediated mitophagy) was assessed. To evaluate the regulation of mitochondrial dynamics, the mRNA level of both *Drp1* (for the fission process) and *Mfn-2* (for the mitochondrial fusion) was assessed.

As ancillary findings, we also considered some morphometric data obtained by quantitative electron microscopy, such as the number of mitochondria for cell section (number of mitochondria/100 μm^2^ of cell section area), the density of mitochondria (mitochondrial total area/100μm^2^ of cell section area), and the density of autophagic vacuoles (AV) (total area of AV/100 μm^2^ of cytoplasmic area).

### TEM and mitochondrial morphometry assessment

PBMCs were washed three-times in PBS and fixed with 2% glutaraldehyde in PBS for 2 h at 4 °C. Samples were post-fixed with 1% osmium tetroxide in veronal acetate buffer pH 7.4 for 1 h at 25 °C, stained with uranyl acetate (5 mg/ml) for 1 h at 25 °C, dehydrated in acetone and embedded in Epon 812 (EMbed 812, Electron Microscopy Science, Hatfield, PA, USA). Ultrathin sections obtained with an Ultracut EMFCS ultramicrotome (Leica Microsystems, Wetzlar, Germany) were unstained or poststained with uranyl acetate and examined under a Morgagni 268D TEM (FEI, Hillsboro, OR, USA) equipped with a Mega View II charge-coupled device camera (SIS, Soft Imaging System GmbH, Munster, Germany).

For the two-dimensional morphometric analysis of mitochondria, at least 20 cell sections in ten different microscopic fields were randomly captured from ultrathin sections of each PBMCs sample and digitalized at 28,000X original magnification. Area of all mitochondria and single cell sections were measured with the AnalySIS software (Soft Imaging System).

### Ultrastructural quantitation of mitochondrial damage

At least 30 cell sections, randomly taken in ten different microscopic fields, obtained from ultrathin sections of each PBMC sample were acquired at 28,000X original magnification and digitalized with a Mega View II charge-coupled device camera (Soft Imaging System).

The micrographs were analyzed with the AnalySIS software (Soft Imaging System) for the percentage of injured mitochondria classified using a grading scale categorized into three levels of damage based on the extension of mitochondrial area provided of intact internal cristae [29, 30]. An additional parameter of the mitochondrial injury was based on the quantitative analysis of convolution degeneration related to the IMM length.

All mitochondria observed in 20 ultrathin sections, randomly taken at 56,000X for all PBMCs samples, were measured for both lengths of IMM and OMM; hence, the IMM/OMM index was calculated for each mitochondrion. Mitochondrial damage was correlated to a low mean value of IMM/OMM index, corresponding to partial or total loss of IMM cristae [31].

### RNA extraction and cDNA synthesis

RNA was extracted using the Quick-RNA^™^ MiniPrep (Zymo Research, Irvine, CA, USA) according to the manufacturer’s instructions. Each sample was treated and quantified, and 1 μg was used to reverse transcription using the iScript cDNA synthesis kit (Bio-Rad) with thermal cycling program as follows: 25 °C for 5 min, followed by 46 °C for 20 min and 95 °C for 1 min.

### Primers

The amplification of the cDNA fragments of target genes expression and of the ribosomal 18S RNA (housekeeping gene) was obtained using specific oligonucleotides chosen through the online tool Primer-BLAST [32] and purchased from Invitrogen (Invitrogen, Carlsbad, CA, USA).

Primers used for the amplification are reported in Table 1. For each primer pair, we performed a no-template control which produced negligible signals.

**Table 1:** Primer sequences used for target and housekeeping genes

Gene	Primer sequence
18S rRNA	5’-CGAGCCGCCTGGATACC-3’ (sense), 5’- CATGGCCTCAGTTCCGAAAA-3’ (antisense);
Beclin-1	5′-GGATGGTGTCTCTCGCAGAT-3′ (sense), 5′-TTTCTTGCCCTTCCTTTCTG- 3′ (antisense)
LC3	5′-CGCACCTTCGAACAAAGAG- 3′ (sense) 5′-TTGGCACTTTCTGTGGACAT- 3′ (antisense)
p66Shc	5’-AATCAGAGAGCCTGCCACATT-3’ (sense) 5’-CTCTTCCTCCTCCTCATC-3’ (antisense);
Parkin	5′-GGCTGTGGGTTTGCCTTCT-3′ (sense), 5′-TGCTTCCCAACGAGCCTG-3′ (antisense)
BNIP3	5′-AGCTCAGACTCTGAGGAAGA-3’ (sense) 5’-CCGACTTGACCAATCCCATATC-3’ (antisense)
FUNDC1	5’-GTGGCTTTCTTCTTCTTCAGATTG-3’ (sense) 5’-CTGCTTTGTTCGCTCGTTTC-3’ (antisense)
Drp1	5’-AATGACCAAGGTGCCTGTAG-3’ (sense) 5’-GCAGTGACAGCGAGGATAAT-3’ (antisense)
Mfn2	5’-GATGGACAGCCCTGGTATTG-3’ (sense) 5’-CACTCACCTTGTGGAAGAAGT-3’ (antisense)

### RT-PCR quantitation

RT-PCR was carried out in 96-well plates using the iQ SYBR Green Supermix (Bio-Rad) and the iCycler Real-Time Detection System (iQ5; Bio-Rad). The thermal cycling program was performed as follows: an initial denaturation step at 95° C for 3 min, followed by 40 cycles at 95 °C for 10 s and 60 °C for 30 s. Melting curves were performed for each primer pair. The relative quantification of gene expression was assessed by comparative threshold cycle method (2^ − ΔΔCT^), including normalization of the gene expression to ribosomal 18S RNA, showing a stable expression in all experimental conditions. All experiments were performed in triplicate and values are reported as mean ± SE.

### Assay of plasma αANP and of NT-proBNP levels

Levels of circulating αANP were assayed by a commercially available ELISA kit (Phoenix Europe GmbH, Karlsruhe, Germany).

NT-proBNP plasma level was determined by an ELISA assay kit (Gruppe Biomedica, Wien, Austria).

### Statistical methods

Student’s *t* test for paired samples was used to evaluate statistical significance among variables with a normal distribution. The values are expressed as mean ± SE. Wilcoxon or Mann–Whitney non-parametric tests were used to compare variables without a normal distribution. *P* values < 0.05 were assumed as statistically significant.

## Results

### Clinical characteristics of the patients

The main clinical, echocardiographic, and laboratory characteristics of the subjects included into the ex vivo and in vivo studies are summarized in Tables 2 and 3.

**Table 2:** Clinical, echocardiographic and laboratory characteristics of chronic HFrEF patients enrolled into the ex vivo and in vivo studies

	*N* = 13 (ex vivo study)	*N* = 6 (in vivo study)
Age (years)	60.1 ± 12.8	61.5 ± 7.7
Male sex (%)	8 (61)	6 (100)
BMI (kg/m^2^)	26.8 ± 3.4	29.8 ± 3.5
NYHA class I (*n*,%)	0	0
NYHA class II (*n*,%)	7 (54)	3 (50)
NYHA class III (*n*,%)	6 (46)	3 (50)
Etiology		
Ischemic (*n*,%)	5 (39)	5 (83)
Idiopathic (*n*,%)	3 (23)	1 (17)
Myocarditis (*n*,%)	2 (15)	0
Valvular heart disease (*n*,%)	3 (23)	0
SBP/DBP (mmHg)	122.6 ± 12.7	124.6 ± 18.1
	75.4 ± 9.8	77.5 ± 8.2
LVEF (%)	38 ± 3.3	33 ± 1.9
LVEDD (mm)	54 ± 7.9	58 ± 4.3
LA size (mm)	36.6 ± 7.7	41.1 ± 6.8
Grade I diastolic dysfunction (*n*,%)	6 (46)	3 (50)
Grade II diastolic dysfunction (*n*,%)	7 (54)	3 (50)
E/e’	9	10
sPAP (mmHg)	33	33
Creatinine (mg/dL)	1.1 ± 0.6	1.0 ± 0.4
BUN (mg/dl)	35.2 ± 18.4	45.9 ± 17.8
Hemoglobin (g/dl) Pharmacological therapy	13.8 ± 1.2	14.7 ± 0.5
ACEi/ARB (*n*,%)	10 (77)	6 (100)*
Beta-blocker (*n*,%)	8 (61)	4 (67)
MRA (*n*,%)	7 (54)	3 (50)
SGLT2i (*n*,%)	0	1 (17)
Loop diuretics (*n*%)	6 (46)	3 (50)
Digoxin (*n*,%)	0	0
Amiodaron (*n*,%)	2 (15)	2 (33)
Ivabradin (*n*,%)	0	1 (17)
Nitrate (*n*,%)	2 (15)	2 (33)

*Before the introduction of the treatment with sacubitril/valsartan

*LVEF* left ventricular ejection fraction, *LVEDD* left ventricular end diastolic diameter, *LA* left atrium, *SBP/DBP* systolic blood pressure/diastolic blood pressure, *sPAP* systolic pulmonary arterial pressure, *ACEi* angiotensin-converting enzyme inhibitor; *ARB* angiotensin receptor blocker; *MRA* mineralocorticoid receptor antagonist; *SGLT2i* sodium–glucose cotransporter 2 inhibitors

**Table 3:** Clinical, echocardiographic and laboratory characteristics of chronic HFeEF patients before and after 2 month treatment with sacubitril/valsartan 97/103 mg twice daily

	HF patients ARNi + (pre-treatment)	HF patients ARNi + (on-treatment)	*P* values
BMI (kg/m^2^)	29.9 ± 3.5	29.8 ± 3.3	NS
NYHA class I (*n*,%)	0 (0)	2(40)	
NYHA class II (*n*,%)	3 (50)	4 (67)	< 0.05
NYHA class III (*n*,%)	3 (50)	2 (33)	
SBP/DBP (mmHg)	124.6 ± 18.1	124.3 ± 17.9	NS
	77.5 ± 8.2	77.3 ± 8.4	
LVEF (%)	33 ± 1.9	38 ± 3.4	< 0.05
LVEDD (mm)	58 ± 4.3	57 ± 4.2	NS
LA size (mm)	41.1 ± 6.8	39.9 ± 7.2	NS
Grade I diastolic dysfunction (*n*,%)	3 (50)	4 (67)	< 0.05
Grade II diastolic dysfunction (*n*,%)	3 (50)	2 (33)	
E/e’	10	7	< 0.05
sPAP (mmHg)	33	30	NS
Creatinine (mg/dL)	1.0 ± 0.4	1.0 ± 0.3	NS
BUN (mg/dl)	45.9 ± 17.8	47.1 ± 18.3	NS
Hemoglobin (g/dl)	14.7 ± 0.5	14.6 ± 0.4	NS

*LVEF* left ventricular ejection fraction, *LVEDD* left ventricular end diastolic diameter, *LA* left atrium, *SBP/DBP* systolic blood pressure/diastolic blood pressure, *sPAP* systolic pulmonary arterial pressure

## Effects of αANP treatment in the ex vivo cultured HF-PBMCs

To evaluate the ex vivo effects exerted by αANP on mitochondrial structure and function, we assessed the values of mitochondrial membrane potential and quantified the ultrastructural changes in mitochondria from PBMCs treated or not with αANP as described in the Methods section.

**Fig. 1:**
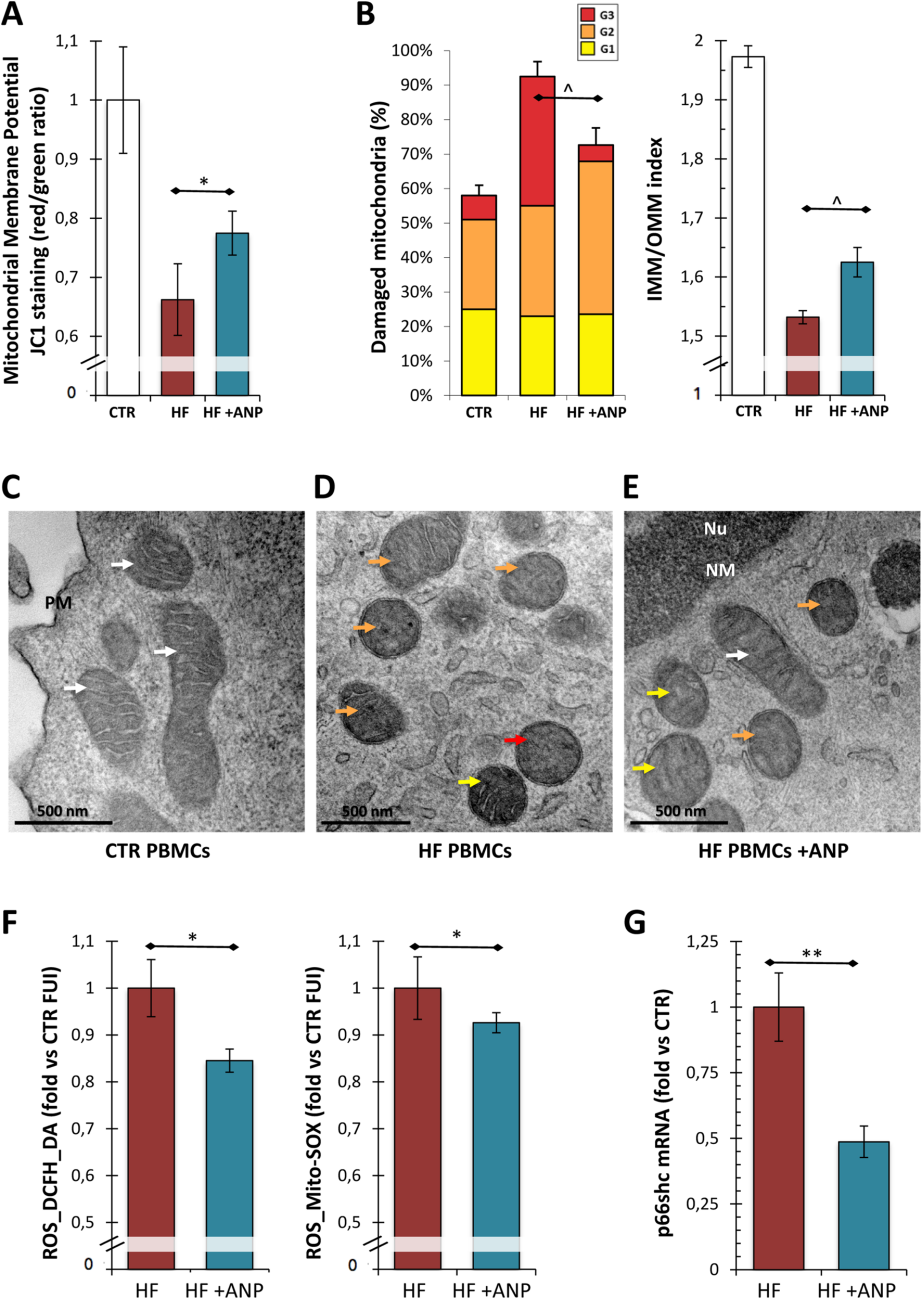
Assessment of mitochondrial function and damage in PBMCs from the ex vivo study. **A** Cytofluorimetric assay for mitochondrial membrane potential ($\Delta \psi {\text{m}}$ ). The JC-1 assay showed significant differences between the PBMCs groups reflecting a mitochondrial depolarization in HF_PBMCs treated or not with αANP (*N* = 10). PBMCs from healthy subjects (*N* = 3) are also shown (CTR). The αANP treatment induced a significant increase of proton motive force in mitochondria from treated HF_PBMCs (Student’s *t* test for paired samples: *p < 0.05 HF vs HF + ANP). **B** Evaluation of mitochondrial ultrastructural damage in CTR_PBMCs and in HF_PBMCs treated or not with αANP. Graphical representation of the ultrastructural damage; the mitochondrial injury was quantified based on the percentage of mitochondrial area displaying intact cristae and of their convolution degree. A severe damage (Mt-G3) and the loss of inner cristae (IMM/OMM index) were higher in the untreated compared to αANP treated HF_PBMCs (*N* = 3; Mann–Whitney test ^*p* = 0.1 for HF vs HF + ANP) and to CTR_PBMCs (*N* = 3) **C**–**E** Representative micrographs of PBMCs mitochondria. Mitochondria of typical shape and size were observed in CTR_PBMCs (see panel **C**). The organelles from untreated HF_PBMCs showed a marked loss of cristae (see panel **D**); in contrast, αANP treated HF_PBMCs (see panel **E**) displayed mitochondria with normal ultrastructural findings or only with a slight damage. (TEM micrographs, uranyl acetate; Legend: *Nu* nucleus; *NM* nuclear membrane, *PM* plasma membrane; Mt-Gx: grade of mitochondrial damage: red arrows: Mt-G3; orange arrows: Mt-G2; yellow arrows: Mt-G1; white arrows: healthy mitochondrion). **F** Assay for the detection of ROS production. The untreated HF_PBMCs generated greater amounts of cytoplasmic (see DCFH_DA probe) and mitochondrial ROS (see Mito-SOX probe) compared with αANP-treated HF_PBMCs (*N* = 10; Student’s *t* test for paired samples: *p < 0.05). **G** In parallel to the decrease of ROS production, αANP downregulated the mRNA expression of *p66shc* gene, a marker of cellular response to oxidative stress (*N* = 5; Wilcoxon test for paired samples: **p < 0.01)

We observed a mitochondrial depolarization in HF_PBMCs compared to PBMCs from healthy subjects (CTR-PBMCs) (Fig. 1A). αANP restored the proton motive force in HF_PBMCs. (Fig. 1A; *p* < 0.05).

We also found a lower mitochondrial damage in CTR_PBMCs with respect to HF_PBMCs (Fig. 1B and Fig. 1C vs Fig. 1D–E). HF_PBMCs showed a burden of damage (Mt-G) higher than in HF_PBMCs treated with αANP (Fig. 1B left panel; *p* = 0.1). Similarly, we observed lower values of the IMM/OMM index in the untreated PBMCs due to the degeneration of convolutions related to lack of the IMM. The αANP treatment promoted the recovery of the IMM extension and determined an improvement of the IMM/OMM index (Fig. 1B right panel; *p* = 0.1; Fig. 1E vs 1D).

Subsequently, to assess whether the positive effects of αANP on restoration of mitochondrial structure and proton motive force observed in the PBMCs could be related to variations in ROS production, we evaluated the total intracellular and mitochondrial ROS levels. In treated PBMCs, the production of ROS was significantly decreased with respect to untreated cells (Fig. 1F; *p* < 0.05). At the same time, by analyzing the mitochondrial stress, we observed that the treatment with αANP induced a down-modulation of *p66shc* mRNA (Fig. 1G; *p* < 0.01). This could result in a reduced cellular response to oxidative stress with subsequent reduction of ROS-induced ROS production and lightening of intracellular oxidative stress load.

**Fig. 2:**
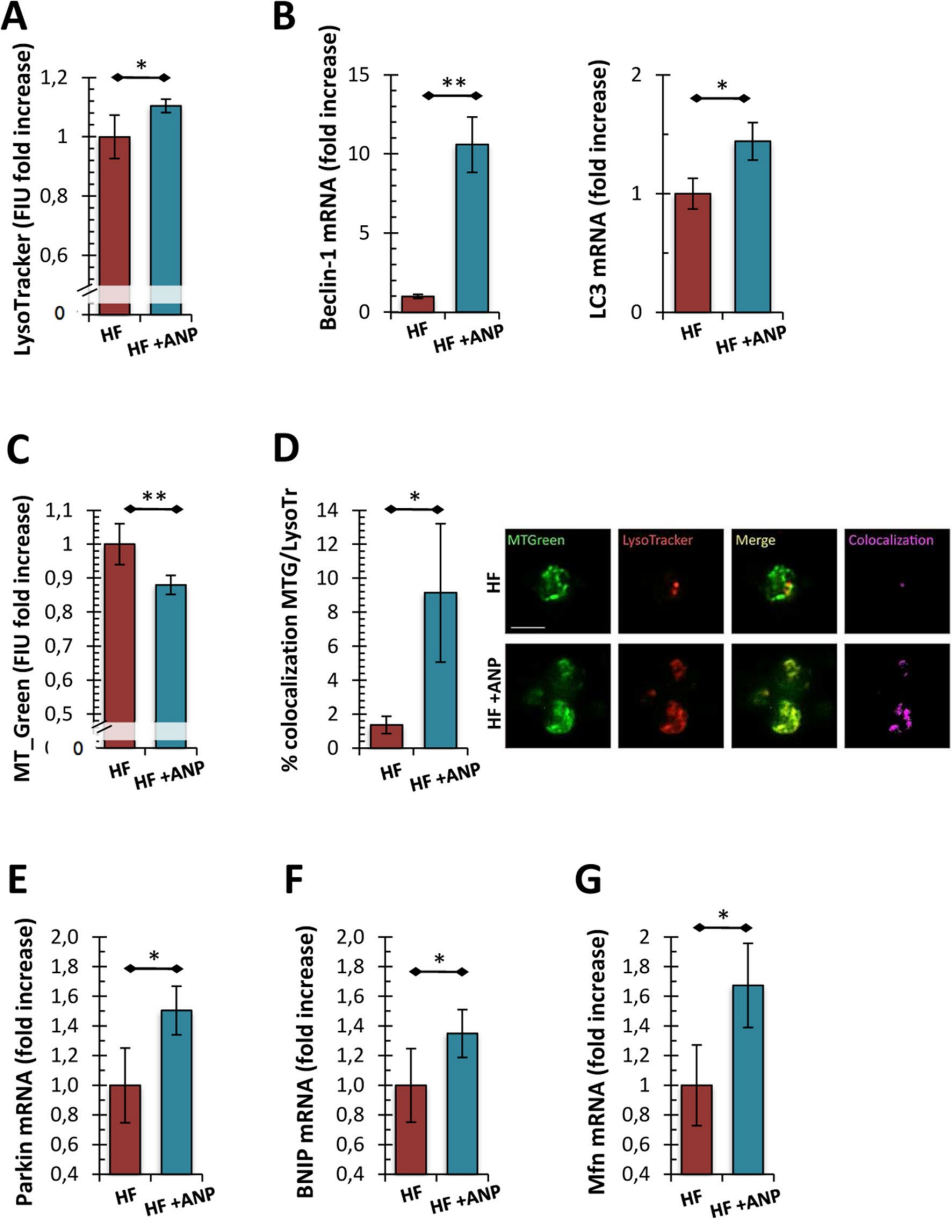
Assessment of autophagy and mitophagy in PBMCs from the ex vivo study. **A** Analysis of acidic compartments by LysoTracker Red probe. The fluorescence intensity levels referred to αANP treated cells were significantly higher than those from the untreated PBMCs (*N* = 10; Student’s *t* test for paired samples: *p < 0.05). **B** Analysis of mRNA expression of genes involved in the autophagic response. *LC3* and *Beclin-1* were upregulated by αANP treatment (*N* = 5; Wilcoxon test for paired samples: *p < 0.05; **p < 0.01). **C** Citofluorimetric assessment of mitochondrial mass. The MitoTracker Green assay showed a net reduction of the mitochondrial mass into αANP treated PBMCs (*N* = 10; see panel **C**; Student’s *t* test for paired samples: **p < 0.01). **D** Analysis of the colocalization signal of mitochondria (in green; MitoTracker) with acidic compartments (in red; LysoTracker) from HF_PBMCs treated or not with αANP. The treatment induced a marked increase of colocalization signal in the treated PBMCs (*N* = 5; in purple; *p < 0.05; Bar: 20 μm). **E**–**G** Analysis of mRNA expression of genes involved in the mitophagic response and in the regulation of mitochondrial dynamics. *Parkin* (**E**), *BNIP3* (**F**) and *Mfn-2* (**G**) were upregulated by αANP treatment (*N* = 5; Wilcoxon test for paired samples: *p < 0.05)

At this point, we attempted to establish whether, even in our experimental conditions, the mitochondrial protective effect induced by αANP treatment was related to the ability to modulate mitochondrial dynamics through an autophagic potentiation mechanism. A significant increase of LysoTracker levels, highlighting a rise of intracellular acidic compartments, was observed after treatment with αANP (Fig. 2A; *p* < 0.05). Alongside the up-modulation of the *LC3* and *Beclin* mRNA levels, two fundamental regulatory genes of autophagy, these data suggested the triggering of a fast and significant autophagic response induced by αANP (Fig. 2B; *p* < 0.01 and *p* < 0.05 respectively). Moreover, the significant reduction in mitochondrial mass was observed in treated PBMCs (Fig. 2C; *p* < 0.01) together with the marked increase of colocalization signal of mitochondrial MitoTracker probe with acidic compartments evoked a stimulation of mitophagy by αANP (Fig. 2D; *p* < 0.05).

The expression of genes related to the mitophagy pathways and to the regulation of mitochondrial dynamics showed an upregulation of *Parkin* and *BNIP3* as well as of *Mfn-2* (Fig. 2E, F and G, respectively; *p* < 0.05). No modulation of *FUNDC2* and *DRP-1* expression was observed (data not shown).

**Fig. 3:**
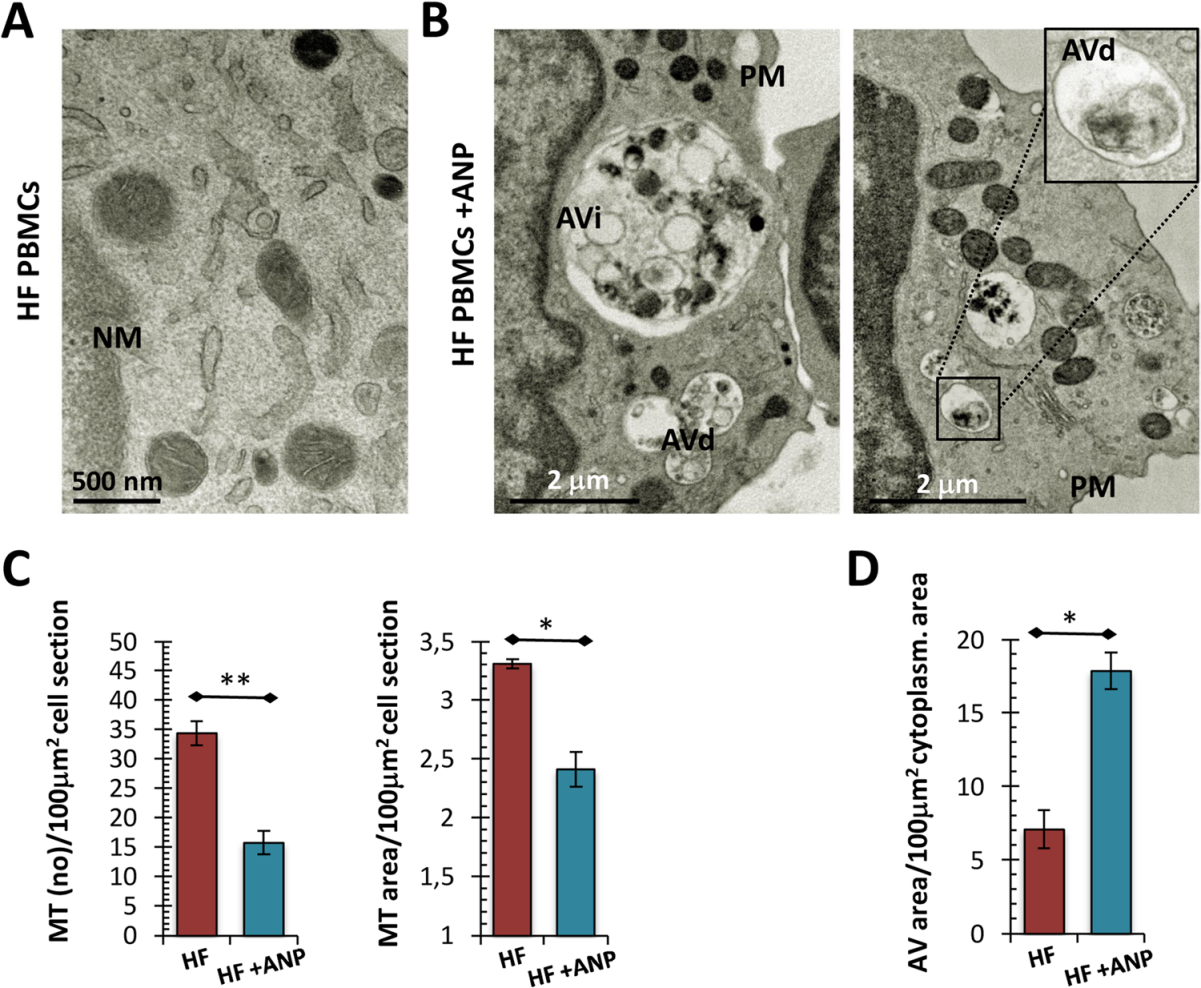
Assessment of autophagy and mitophagy in PBMCs from the ex vivo study (2): ultrastructural morphometric analysis. **A**, **B** Representative micrographs of PBMCs mitochondria. The ultrastructural analysis showed an extensive activation of autophagic process in treated cells. Unlike untreated PBMCs (**A**), several degradative autophagic vacuoles were identifiable into the cytoplasm (**B**). Partially degraded mitochondria filled a significant part of autophagosomes (see magnification). (TEM micrographs, uranyl acetate; Abbreviations: *NM* nuclear membrane; *PM* plasma membrane; *AVi* early autophagic vacuole; *AVd* degradative autophagic vacuole). **C**, **D** Morphometric data related to intracellular density of mitochondria and of AV. Consistently with what observed in the cytofluorimetric assay, the number of mitochondria for cell section and the intracellular density of mitochondria were significantly lower in treated PBMCs (*N* = 5; see panel **C**; Student’s *t* test for paired samples: **p < 0.01 and *p < 0.05 respectively). Furthermore, the treated HF_PBMCs showed a significant increase in the amount of intracytoplasmic autophagic structures respect to untreated PBMCs (D) (*N* = 5; Student’s *t* test for paired samples: *p < 0.05)

We also confirmed by TEM analysis that the treated PBMCs had less mitochondrial mass than untreated PBMCs, presenting both a lower number of mitochondrial structures and a smaller cell surface occupied by mitochondria (Fig. 3A–C; *p* < 0.01 and *p* < 0.05 respectively). In addition, the cells treated with αANP showed a greater number of voluminous early and late autophagic structures, also containing partially degraded mitochondria, within the cytoplasm respect to untreated cells. The latter displayed only rarely small autophagic vacuoles in their cytoplasm (Fig. 3A–B; Fig. 3D; *p* < 0.05).

### In vivo effects of ARNi treatment on HF-PBMCs

In a separate set of experiments, we studied the mitochondrial performances, the oxidative stress levels, and the activation of the autophagy/mitophagy pathways in PBMCs isolated from HF patients before and after two-month therapy with sacubitril/valsartan, a treatment able to activate the αANP level due to neprilysin inhibition [20, 21, 33].

**Fig. 4:**
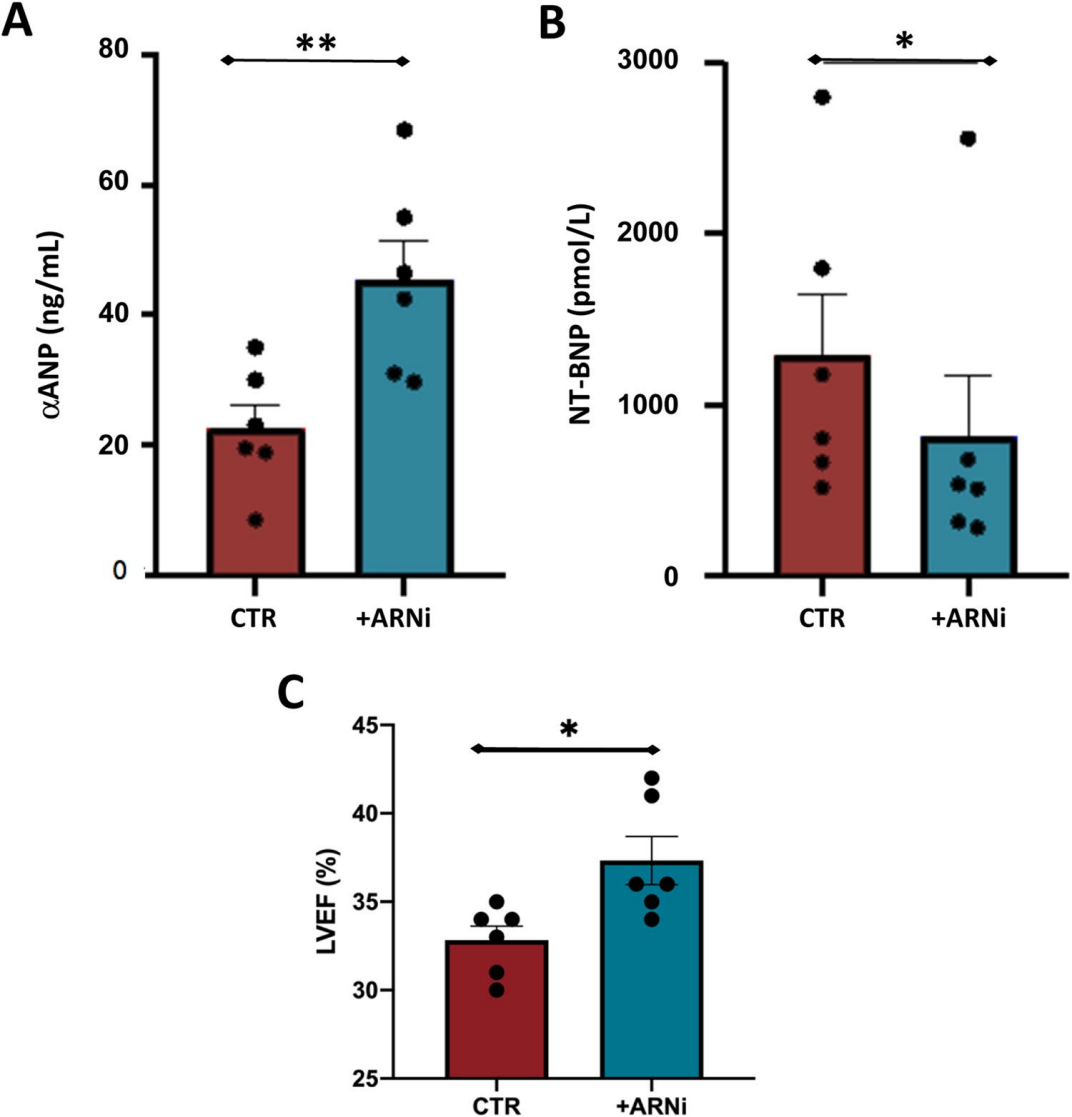
Impact of ARNi on plasma levels of both αANP and NT-BNP and on LVEF. The evaluations were performed at baseline (CTR) and after 2 months of treatment (+ ARNi). **A** αANP; **B** NT-BNP; **C** LVEF (*N* = 6 for HF group; Student’s *t* test for paired samples *p < 0.05)

To establish the effect of ARNi on both the metabolism of αANP and the ventricular function, we measured the plasma levels of both αANP and NT-proBNP before and after treatment. As expected, after pharmacological treatment, we observed an increase in plasma levels of αANP (Fig. 4A; *p* < 0.01). At the same time, because of the improvement in cardiac function, we observed a significant reduction of NT-proBNP plasma levels (Fig. 4B; *p* < 0.05) in agreement with the improvement of LVEF documented by echocardiography (Fig. 4C; Table 2; *p* < 0.05).

**Fig. 5:**
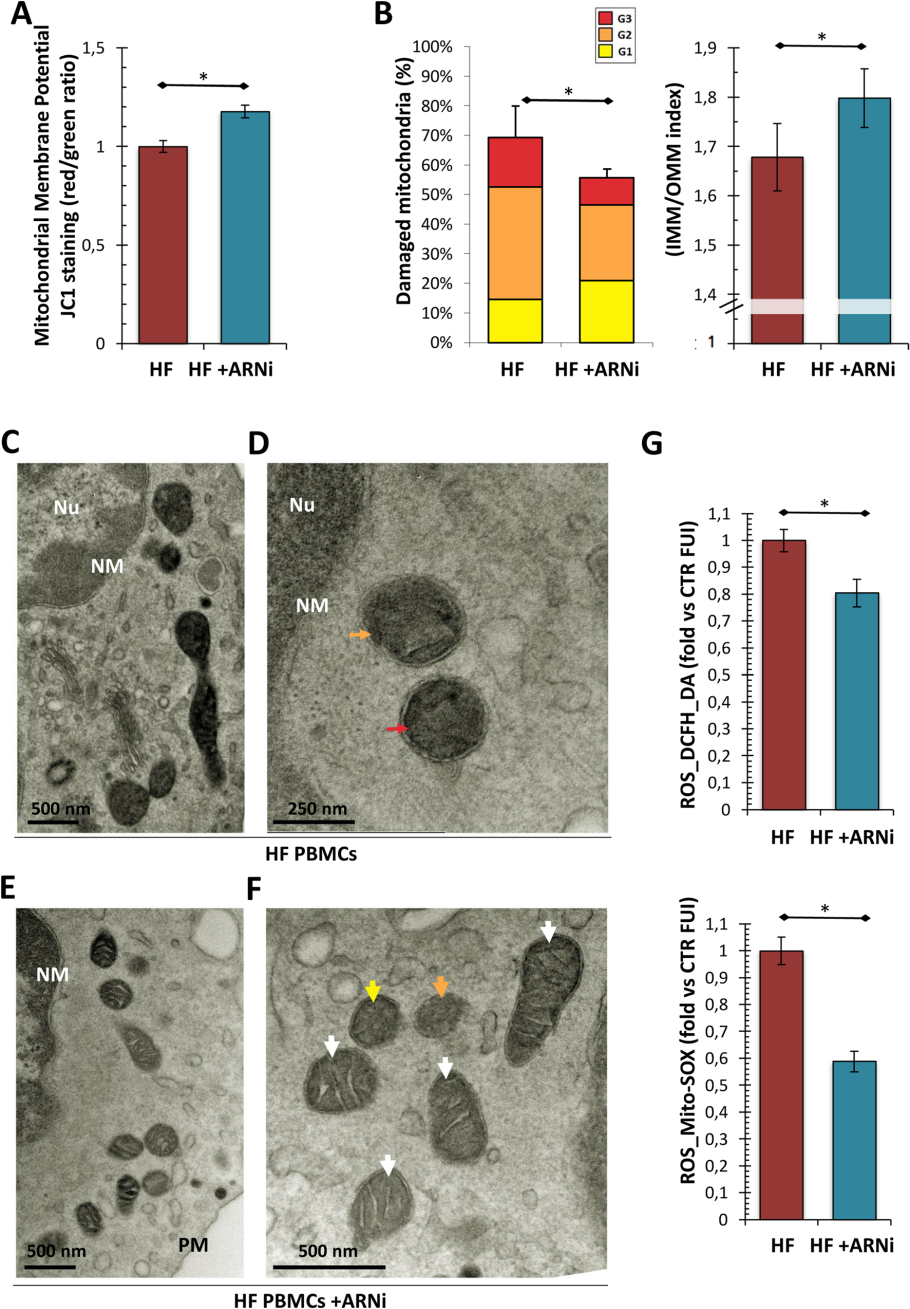
Assessment of mitochondrial function and damage in PBMCs from the in vivo study. **A** Cytofluorimetric assay for mitochondrial membrane potential (JC-1 staining) in PBMCs isolated from patients before and after ARNi treatment. The data reflect a significant mitochondrial repolarization after ARNi treatment (*N* = 6; Student’s *t* test for paired samples: *p < 0.05). **B** Graphical representation of the ultrastructural mitochondrial grading of damage. The burden of overall damage was lower after ARNi treatment (*N* = 6; Student’s *t* test for paired samples: *p < 0.05). Similarly, the IMM/OMM index associated with convolution loss of inner mitochondrial membrane was lower in the PBMCs isolated before the treatment (*N* = 6; Student’s *t* test for paired samples: *p < 0.05) **C**–**F** Representative micrographs of ultrastructural damage in different PBMC samples. The burden of mitochondrial damage was higher in PBMCs obtained before the starting of therapy; the mitochondria were characterized by degeneration of convolutions related to lack of the inner membrane and accordingly by reduction of mitochondrial area with intact cristae (see **C** and **D** panels). After the ARNi treatment, the PBMCs showed mitochondria with normal morphology or with slight damage (see **E** and **F** panels). (TEM micrographs, uranyl acetate/lead citrate; Legend: *Nu* nucleus; *NM* nuclear membrane, *PM* plasma membrane; Mt-Gx: grade of mitochondrial damage: red arrows: Mt-G3; orange arrows: Mt-G2; yellow arrows: Mt-G1; white arrows: healthy mitochondrion). **G** Cytofluorimetric assay for the evaluation of cytoplasmic (see DCFH_DA probe) and mitochondrial ROS (see Mito-SOX probe) generation. The cytoplasmic and mitochondrial ROS levels were lower in PBMCs after ARNi treatment (Student’s *t* test for paired samples: *p < 0.05)

After collecting these preliminary data, we proceeded to evaluate the mitochondrial dynamics in the PBMCs of the treated patients. The comparison for paired data showed that the PBMCs collected after treatment were characterized by mitochondria with better functional performances (Fig. 5A–F; *p* < 0.05) provided of more intact ultrastructure (Fig. 5E–F) and producing lower mitochondrial ROS levels (Fig. 5G; *p* < 0.05).

**Fig. 6:**
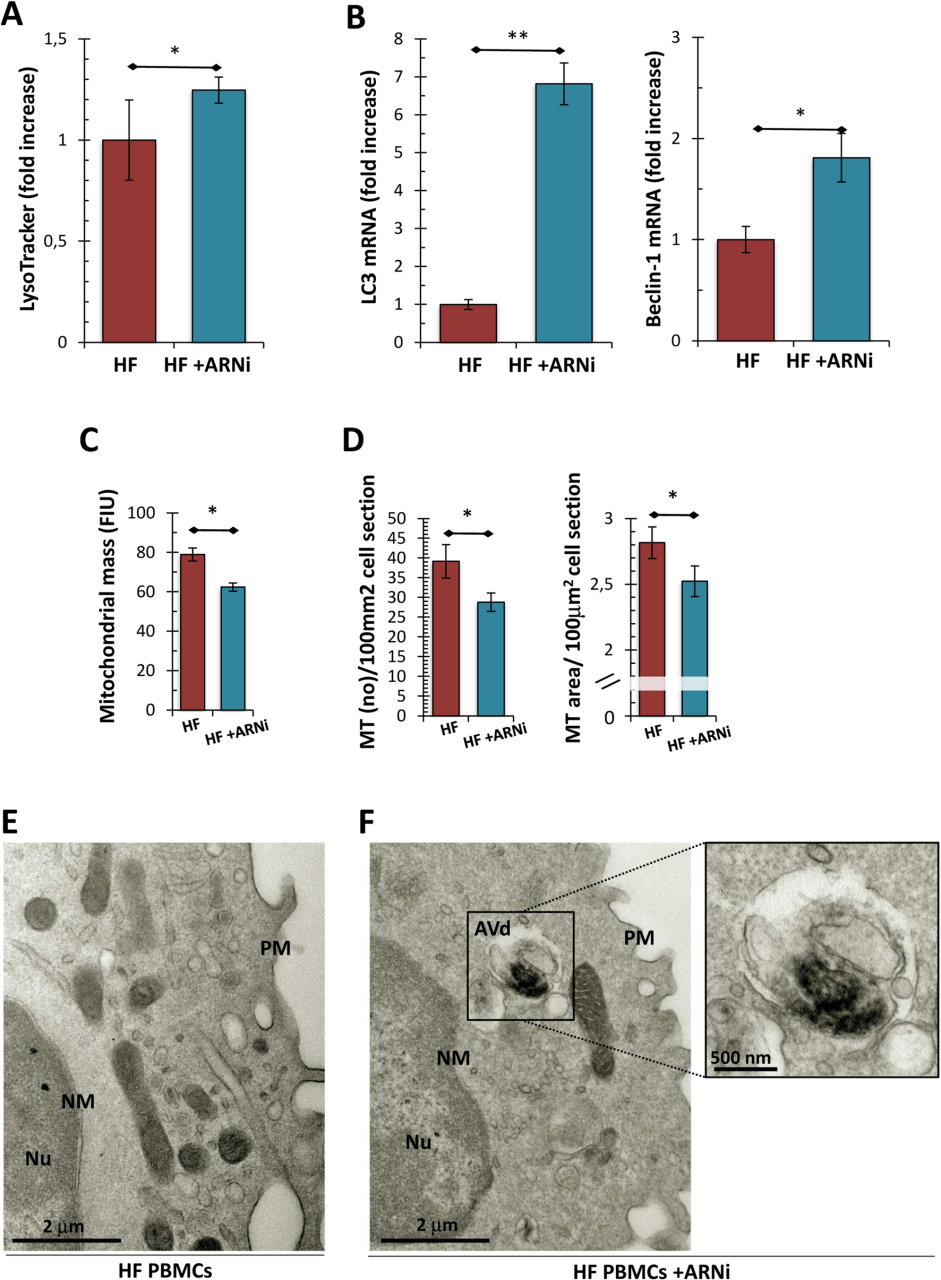
Assessment of autophagy and mitophagy in PBMCs from the in vivo study. **A** Analysis of acidic compartments by LysoTracker Red probe. The fluorescence intensity levels referred to PBMCs obtained after ARNi treatment are higher than those recorded in the same patients before starting therapy (*N* = 6; Wilcoxon test for paired samples: *p < 0.05). **B** Analysis of mRNA expression of genes involved in the autophagic response. *LC3* and *beclin* were upregulated by ARNi (*N* = 5 for HF group; *N* = 3 for CTR; Wilcoxon test for paired samples: *p < 0.05; **p < 0.01). **C**, **D** Assessment of mitochondrial mass. The MitoTracker Green assay showed an evident decrease of the mitochondrial mass into PBMCs from treated patients (*N* = 10; see panel **C**; Wilcoxon test for paired samples: *p < 0.05). These data were corroborated by the ultrastructural morphometric analysis. The number of mitochondria for cell section and the mitochondrial density were significantly lower in PBMCs isolated after therapy (*N* = 10; see panel D; Student’s *t* test for paired samples: **p < 0.01 and *p < 0.05 respectively). **E**–**F** Representative micrographs of PBMCs obtained from patients before and after ARNi therapy. The ultrastructural analysis confirmed the activation of autophagy after ARNi treatment: degradative compartments with degraded mitochondrial structures are recognizable into the cytoplasm of PBMCs from treated patients (see magnification in panel **F**). [(TEM micrographs, uranyl acetate; Abbreviations: *NM* nuclear membrane; *PM* plasma membrane; *AVi* early autophagic vacuole; *AVd* degradative autophagic vacuole)]

As expected, the treatment appeared to modulate the activation of the autophagic pathway. In fact, the PBMCs of the treated patients showed an increased level of acid compartments (Fig. 6A; *p* < 0.05) associated with an up-modulation of *LC3* and *Beclin* mRNA levels (Fig. 6B; *p* < 0.01 and *p* < 0.05, respectively) and a reduction of mitochondrial mass (Fig. 6C and D; *p* < 0.05). Also in this experimental context, the activation of autophagy was confirmed by ultrastructural evaluation that showed a marked increase in the amount of intracytoplasmic AV (Fig. 6E and F).

## Discussion

In the present study, we demonstrate for the first time the ability of αANP to counteract the mitochondrial dysfunction through a strengthening of the autophagic response in a clinical setting of HFrEF patients. We based our experimental system on the analysis of PBMCs, an easily available biological matrix with a consolidated reliability in the pathophysiological studies of cardiovascular diseases including HF [34].

Mitochondrial oxidative phosphorylation is an essential requirement for the metabolic supplies of the cardiomyocytes, and a quality control of the mitochondrial network is crucial to ensure the functional performance of the myocardial tissue [35].

Mitochondrial homeostasis is regulated by specific mechanisms that preserve organelle structure and performance. Therefore, the maintenance of an adequate pool of mitochondria is constantly adapted by mitochondrial dynamics to changes within the cellular microenvironment through the selective elimination of the damaged organelles by mitophagy [36, 37].

In cardiac diseases, mitochondrial dysfunction contributes to uncoupling of the OXPHOS, limits the energy production, increases ROS generation, and delivers apoptotic signals [38]. These events exacerbate mitochondrial damage by creating a vicious circle leading to a pro-apoptotic, pro-fibrotic, and hypo-contractile histopathological phenotypes [39, 40].

Over the last decades, the scientific community has repeatedly attempted to develop biomarkers useful to monitor the oxidative stress to gain information on the state and progression of the cardiovascular disease, also in response to different or innovative therapeutic strategies [41]. Among the most promising experimental tools, the isolation and the analysis of PBMCs represent a suitable and non-invasive approach to measure both oxidative stress and mitochondrial functionality in vivo. To date, this strategy has been investigated in some pathological settings, such as hypertension, atherosclerotic and ischemic heart disease, stroke, and HF [34].

In HF patients, PBMCs undergo functional and structural mitochondrial changes like those of the failing cardiomyocytes [42]. Furthermore, studies on leukocyte mitochondria in advanced and decompensated congestive HF or after cardiac surgery showed that the degree of mitochondrial dysfunction of PBMCs is related to the disease severity and that this alteration gradually recovers after clinical stabilization [43, 44]. The rationale for these observations is based on the hypothesis that circulating leukocytes, free to flow incessantly in the blood stream, may reflect quite closely both metabolic state and oxidative stress status of the myocardial tissue. In addition, the dysfunctional PBMCs can jeopardize the functional homeostasis of the target tissue in which they recirculate, including the myocardial tissue, through the discharge of huge amounts of ROS [45].

We recently showed that circulating leucocytes from HFrEF patients can generate a state of oxidative stress because they contain structurally and functionally altered mitochondria due to an inability to activate an effective antioxidant response and an adequate mitophagic flux [16]. Therefore, strategies able to stimulate autophagy/mitophagy and to improve mitochondrial function may represent an adequate approach for the treatment of HF.

ANP, secreted by cardiomyocytes in response to stressors such as pressure or volume overload, is known to exert several protective functions within the cardiovascular system by opposing cardiomyocyte hypertrophy, reducing cardiac fibrosis, and promoting vascular integrity. A novel property of αANP is the promotion and regulation of cardiac autophagy. In fact, we recently demonstrated that αANP exerts a cardioprotective effect through the NPR-A/PKG/TFEB signaling pathway regulating autophagy both in vitro and in vivo [11]. This hormone also protects mitochondrial structure and function in other experimental models [46].

Based on these premises, it becomes interesting to verify whether αANP could promote autophagy/mitophagy and could ameliorate mitochondrial damage and function in chronic HF patients using the isolated PBMCs.

The double approach applied in our experimental design, including both ex vivo and in vivo studies, confirmed our hypothesis. The ex vivo study directly demonstrated the ability of physiological concentrations of αANP to stimulate autophagy/mitophagy, to ameliorate mitochondrial damage and function, and to reduce oxidative stress generation. The *parkin* upregulation observed in treated PBMCs supports the activation of the ubiquitin-dependent PINK1-/Parkin-mediated mitophagy which, as known, works to ensure the clearance of depolarized mitochondria from cells [47]. In this context, the up-modulation of *BNIP*, whose role must necessarily be clarified by further mechanistic studies, could have a further negative impact on the membrane potential of already damaged mitochondria favoring their removal through a Parkin-mediated mitophagy [48]. Finally, the upregulation of *Mfn-2* could support the evidence of an effective mitochondrial network restoration induced by αANP [49].

All these data indicate that αANP ameliorates the morpho-functional hallmarks in the ex vivo setting using PBMCs isolated from HF patients. In fact, αANP treatment dampened the mitochondrial depolarization and the ultrastructural damage therefore counteracting the mitochondrial oxidative stress. Similar findings were obtained with the in vivo approach through the administration of sacubitril/valsartan. In this regard, we cannot exclude that the drug may have exerted its effects, at least in part, through other nonspecific mechanisms and in concert with the conventional drugs administered to patients.

Taken together, our results indicate that the ex vivo exposure to αANP in an experimental system based on cultures of PBMCs isolated from HF patients exerts beneficial cellular effects by reducing mitochondrial dysfunction and the related oxidative stress through autophagy activation, supporting the recent evidence obtained in other experimental models [11]. Although the effects of αANP observed in the PBMC might not entirely result in similar beneficial effects in the cardiac cells/tissue, the data obtained support the benefit of this therapy as a fundamental strategy to enhance, through neprilysin inhibition, the endogenous αANP level and its protective effects.

A limitation of the current investigation is represented by the small number of HF patients enrolled in the in vivo study, mainly because of the highly selected study criteria. On the other hand, by borrowing an approach now widely used in the oncology field, the development of liquid biopsy techniques for the study of PBMCs has the credentials to become a practical study tool in the translational research of cardiovascular diseases.

In conclusion, our current original study reinforces αANP as a key hormone for the treatment of HF not only for its well-known hemodynamic properties but also for its protective autocrine cellular functions. In particular, the novelty of our work, supporting the efficacy of αANP as a cardioprotective hormone capable of restoring adequate mitochondrial structure and function by modulating the autophagy/mitophagy processes, provides new mechanistic insights into the therapeutic action of sacubitril/valsartan in HFrEF patients.

## Data Availability

The datasets generated during the current study are available from the corresponding author on reasonable request.

## Abbreviations

ANP: Atrial natriuretic peptide
NT-proBNP: Amino terminal brain natriuretic peptide
ARNi: Angiotensin receptor/neprilysin inhibition
AV: Autophagic vacuoles
BNIP3: BCL2-interacting protein 3
CTR: Control
Drp1: Dynamin-related protein 1
FUNDC1: FUN14 domain-containing protein 1
IMM: Inner mitochondrial membrane
HF: Heart failure
HFrEF: Heart failure with reduced ejection fraction
Mfn-2: Mitofusin 2
NPRA: Type A natriuretic peptide receptor
OXPHOS: Oxidative phosphorylation system
OMM: Outer mitochondrial membrane
PKG: Protein kinase G
PBMCs: Peripheral blood mononuclear cells
ROS: Reactive oxygen species
TEM: Transmission electron microscopy
TFEB: Transcription factor EB

## References

[1] GBD (2017). Disease and Injury Incidence and Prevalence Collaborators (2018) Global, regional, and national incidence, prevalence, and years lived with disability for 354 diseases and injuries for 195 countries and territories, 1990–2017: a systematic analysis for the Global Burden of Disease Study 2017. Lancet.

[2] Grieve DJ, Shah AM (2003). Oxidative stress in heart failure. More than just damage. Eur Heart J.

[3] Rosca MG, Hoppel CL (2013). Mitochondrial dysfunction in heart failure. Heart Fail Rev.

[4] Redout EM, Wagner MJ, Zuidwijk MJ, Boer C, Musters RJ, van Hardeveld C, Paulus WJ, Simonides WS (2007). Right-ventricular failure is associated with increased mitochondrial complex II activity and production of reactive oxygen species. Cardiovasc Res.

[5] Kaludercic N, Takimoto E, Nagayama T, Feng N, Lai EW, Bedja D, Chen K, Gabrielson KL, Blakely RD, Shih JC, Pacak K, Kass DA, Di Lisa F, Paolocci N (2010). Monoamine oxidase A-mediated enhanced catabolism of norepinephrine contributes to adverse remodeling and pump failure in hearts with pressure overload. Circ Res.

[6] Hayashi D, Ohshima S, Isobe S, Cheng XW, Unno K, Funahashi H, Shinoda N, Okumura T, Hirashiki A, Kato K, Murohara T (2013). Increased (99m)Tc-sestamibi washout reflects impaired myocardial contractile and relaxation reserve during dobutamine stress due to mitochondrial dysfunction in dilated cardiomyopathy patients. J Am Coll Cardiol.

[7] Ahuja P, Wanagat J, Wang Z, Wang Y, Liem DA, Ping P, Antoshechkin IA, Margulies KB, Maclellan WR (2013). Divergent mitochondrial biogenesis responses in human cardiomyopathy. Circulation.

[8] Rubattu S, Volpe M (2019). Natriuretic peptides in the cardiovascular system: multifaceted roles in physiology, pathology and therapeutics. Int J Mol Sci.

[9] Volpe M, Gallo G, Rubattu S (2023). Endocrine functions of the heart: from bench to bedside. Eur Heart J.

[10] Forte M, Madonna M, Schiavon S, Valenti V, Versaci F, Zoccai GB, Frati G, Sciarretta S (2019). Cardiovascular pleiotropic effects of natriuretic peptides. Int J Mol Sci.

[11] Forte M, Marchitti S, Di Nonno F, Stanzione R, Schirone L, Cotugno M, Bianchi F, Schiavon S, Raffa S, Ranieri D, Fioriniello S, Della Ragione F, Torrisi MR, Carnevale R, Valenti V, Versaci F, Frati G, Vecchione C, Volpe M, Rubattu S, Sciarretta S (2023). NPPA/atrial natriuretic peptide is an extracellular modulator of autophagy in the heart. Autophagy.

[12] Levine B, Kroemer G (2008). Autophagy in the pathogenesis of disease. Cell.

[13] Feng Y, He D, Yao Z, Klionsky DJ (2014). The machinery of macroautophagy. Cell Res.

[14] Saito T, Sadoshima J (2015). Molecular mechanisms of mitochondrial autophagy/mitophagy in the heart. Circ Res.

[15] Saito T, Hamano K, Sadoshima J (2020). Molecular mechanisms and clinical implications of multiple forms of mitophagy in the heart. Cardiovasc Res.

[16] Coluccia R, Raffa S, Ranieri D, Micaloni A, Valente S, Salerno G, Scrofani C, Testa M, Gallo G, Pagannone E, Torrisi MR, Volpe M, Rubattu S (2018). Chronic heart failure is characterized by altered mitochondrial function and structure in circulating leucocytes. Oncotarget.

[17] Sciarretta S, Zhai P, Volpe M, Sadoshima J (2012). Pharmacological modulation of autophagy during cardiac stress. J Cardiovasc Pharmacol.

[18] Shirakabe A, Ikeda Y, Sciarretta S, Zablocki DK, Sadoshima J (2016). Aging and autophagy in the heart. Circ Res.

[19] Vasquez N, Carter S, Grodin JL (2020). Angiotensin receptor-neprilysin inhibitors and the natriuretic peptide axis. Curr Heart Fail Rep.

[20] Nougué H, Pezel T, Picard F, Sadoune M, Arrigo M, Beauvais F, Launay JM, Cohen-Solal A, Vodovar N, Logeart D (2019). Effects of sacubitril/valsartan on neprilysin targets and the metabolism of natriuretic peptides in chronic heart failure: a mechanistic clinical study. Eur J Heart Fail.

[21] Ibrahim NE, McCarthy CP, Shrestha S, Gaggin HK, Mukai R, Szymonifka J, Apple FS, Burnett JC, Iyer S, Januzzi JL (2019). Effect of neprilysin inhibition on various natriuretic peptide assays. J Am Coll Cardiol.

[22] Klionsky DJ (2021). Guidelines for the use and interpretation of assays for monitoring autophagy (4th edition). Autophagy.

[23] Kaludercic N, Maiuri MC, Kaushik S, Fernández ÁF, de Bruijn J, Castoldi F, Chen Y, Ito J, Mukai R, Murakawa T, Nah J, Pietrocola F, Saito T, Sebti S, Semenzato M, Tsansizi L, Sciarretta S, Madrigal-Matute J (2020). Comprehensive autophagy evaluation in cardiac disease models. Cardiovasc Res.

[24] Chikte S, Panchal N, Warnes G (2014). Use of LysoTracker dyes: a flow cytometric study of autophagy. Cytometry A.

[25] Ricci A, Cherubini E, Scozzi D, Pietrangeli V, Tabbì L, Raffa S, Leone L, Visco V, Torrisi MR, Bruno P, Mancini R, Ciliberto G, Terzano C, Mariotta S (2013). Decreased expression of autophagic beclin 1 protein in idiopathic pulmonary fibrosis fibroblasts. J Cell Physiol.

[26] Mizushima N, Yoshimori T, Levine B (2010). Methods in mammalian autophagy research. Cell.

[27] Warnes G (2015). Flow cytometric assays for the study of autophagy. Methods.

[28] Forte M, Marchitti S, Cotugno M, Di Nonno F, Stanzione R, Bianchi F, Schirone L, Schiavon S, Vecchio D, Sarto G, Scioli M, Raffa S, Tocci G, Relucenti M, Torrisi MR, Valenti V, Versaci F, Vecchione C, Volpe M, Frati G, Rubattu S, Sciarretta S (2021). Trehalose, a natural disaccharide, reduces stroke occurrence in the stroke-prone spontaneously hypertensive rat. Pharmacol Res..

[29] Putignani L, Raffa S, Pescosolido R, Rizza T, Del Chierico F, Leone L, Aimati L, Signore F, Carrozzo R, Callea F, Torrisi MR, Grammatico P (2012). Preliminary evidences on mitochondrial injury and impaired oxidative metabolism in breast cancer. Mitochondrion.

[30] Raffa S, Scrofani C, Valente S, Micaloni A, Forte M, Bianchi F, Coluccia R, Geurts AM, Sciarretta S, Volpe M, Torrisi MR, Rubattu S (2017). In vitro characterization of mitochondrial function and structure in rat and human cells with a deficiency of the NADH: ubiquinone oxidoreductase Ndufc2 subunit. Hum Mol Genet.

[31] Raffa S, Chin XLD, Stanzione R, Forte M, Bianchi F, Cotugno M, Marchitti S, Micaloni A, Gallo G, Schirone L, Tocci G, Violini R, Torrisi MR, Volpe M, Rubattu S (2019). The reduction of NDUFC2 expression is associated with mitochondrial impairment in circulating mononuclear cells of patients with acute coronary syndrome. Int J Cardiol.

[32] Ye J, Coulouris G, Zaretskaya I, Cutcutache I, Rozen S, Madden TL (2012). Primer-BLAST: a tool to design target-specific primers for polymerase chain reaction. BMC Bioinformatics.

[33] Rubattu S, Cotugno M, Forte M, Stanzione R, Bianchi F, Madonna M, Marchitti S, Volpe M (2018). Effects of dual angiotensin type 1 receptor/neprilysin inhibition vs. angiotensin type 1 receptor inhibition on target organ injury in the stroke-prone spontaneously hypertensive rat. J Hypertens.

[34] Rubattu S, Forte M, Raffa S (2019). Circulating leukocytes and oxidative stress in cardiovascular diseases: a state of the art. Oxid Med Cell Longev.

[35] Vásquez-Trincado C, García-Carvajal I, Pennanen C, Parra V, Hill JA, Rothermel BA, Lavandero S (2016). Mitochondrial dynamics, mitophagy and cardiovascular disease. J Physiol.

[36] Forte M, Schirone L, Ameri P, Italian Society of Cardiology Working group on Cellular and Molecular Biology of the Heart (2012). The role of mitochondrial dynamics in cardiovascular diseases. Br J Pharmacol.

[37] Ikeda Y, Sciarretta S, Nagarajan N, Rubattu S, Volpe M, Frati G, Sadoshima J (2014). New insights into the role of mitochondrial dynamics and autophagy during oxidative stress and aging in the heart. Oxid Med Cell Longev.

[38] Zorov DB, Juhaszova M, Sollott SJ (2014). Mitochondrial reactive oxygen species (ROS) and ROS-induced ROS release. Physiol Rev.

[39] Calvieri C, Rubattu S, Volpe M (2012). Molecular mechanisms underlying cardiac antihypertrophic and antifibrotic effects of natriuretic peptides. J Mol Med (Berl).

[40] Cannavo A, Komici K, Bencivenga L, D'amico ML, Gambino G, Liccardo D, Ferrara N, Rengo G (2018). GRK2 as a therapeutic target for heart failure. Expert Opin Ther Targets.

[41] Marrocco I, Altieri F, Peluso I (2017). Measurement and clinical significance of biomarkers of oxidative stress in humans. Oxid Med Cell Longev.

[42] Song B, Li T, Chen S, Yang D, Luo L, Wang T, Han X, Bai L, Ma A (2016). Correlations between MTP and ROS levels of peripheral blood lymphocytes and readmission in patients with chronic heart failure. Heart Lung Circ.

[43] Kong CW, Hsu TG, Lu FJ, Chan WL, Tsai K (2001). Leukocyte mitochondria depolarization and apoptosis in advanced heart failure: clinical correlations and effect of therapy. J Am Coll Cardiol.

[44] Kong CW, Huang CH, Hsu TG, Tsai KK, Hsu CF, Huang MC, Chen LC (2004). Leukocyte mitochondrial alterations after cardiac surgery involving cardiopulmonary bypass: clinical correlations. Shock.

[45] Ijsselmuiden AJ, Musters RJ, de Ruiter G, van Heerebeek L, Alderse-Baas F, van Schilfgaarde M, Leyte A, Tangelder GJ, Laarman GJ, Paulus WJ (2008). Circulating white blood cells and platelets amplify oxidative stress in heart failure. Nat Clin Pract Cardiovasc Med.

[46] Oi Y, Nagoshi T, Kimura H, Tanaka Y, Yoshii A, Yasutake R, Takahashi H, Kashiwagi Y, Tanaka TD, Tachibana T, Yoshimura M (2022). Exogenous ANP treatment ameliorates myocardial insulin resistance and protects against ischemia-reperfusion injury in diet-induced obesity. Int J Mol Sci.

[47] Lee Y, Lee HY, Hanna RA, Gustafsson ÅB (2011). Mitochondrial autophagy by Bnip3 involves Drp1-mediated mitochondrial fission and recruitment of Parkin in cardiac myocytes. Am J Physiol Heart Circ Physiol.

[48] Narendra D, Tanaka A, Suen DF, Youle RJ (2008). Parkin is recruited selectively to impaired mitochondria and promotes their autophagy. J Cell Biol.

[49] López-Doménech G, Howden JH, Covill-Cooke C, Morfill C, Patel JV, Bürli R, Crowther D, Birsa N, Brandon NJ, Kittler JT (2021). Loss of neuronal Miro1 disrupts mitophagy and induces hyperactivation of the integrated stress response. EMBO J..

